# DNA Barcode Libraries Provide Insight into Continental Patterns of Avian Diversification

**DOI:** 10.1371/journal.pone.0020744

**Published:** 2011-07-27

**Authors:** Darío A. Lijtmaer, Kevin C. R. Kerr, Ana S. Barreira, Paul D. N. Hebert, Pablo L. Tubaro

**Affiliations:** 1 División Ornitología, Museo Argentino de Ciencias Naturales “Bernardino Rivadavia,” Buenos Aires, Argentina; 2 Department of Integrative Biology, Biodiversity Institute of Ontario, University of Guelph, Guelph, Ontario, Canada; American Museum of Natural History, United States of America

## Abstract

**Background:**

The causes for the higher biodiversity in the Neotropics as compared to the Nearctic and the factors promoting species diversification in each region have been much debated. The refuge hypothesis posits that high tropical diversity reflects high speciation rates during the Pleistocene, but this conclusion has been challenged. The present study investigates this matter by examining continental patterns of avian diversification through the analysis of large-scale DNA barcode libraries.

**Methodology and Principal Findings:**

Standardized COI datasets from the avifaunas of Argentina, the Nearctic, and the Palearctic were analyzed. Average genetic distances between closest congeners and sister species were higher in Argentina than in North America reflecting a much higher percentage of recently diverged species in the latter region. In the Palearctic genetic distances between closely related species appeared to be more similar to those of the southern Neotropics. Average intraspecific variation was similar in Argentina and North America, while the Palearctic fauna had a higher value due to a higher percentage of variable species. Geographic patterning of intraspecific structure was more complex in the southern Neotropics than in the Nearctic, while the Palearctic showed an intermediate level of complexity.

**Conclusions and Significance:**

DNA barcodes can reveal continental patterns of diversification. Our analysis suggests that avian species are older in Argentina than in the Nearctic, supporting the idea that the greater diversity of the Neotropical avifauna is not caused by higher recent speciation rates. Species in the Palearctic also appear to be older than those in the Nearctic. These results, combined with the patterns of geographic structuring found in each region, suggest a major impact of Pleistocene glaciations in the Nearctic, a lesser effect in the Palearctic and a mild effect in the southern Neotropics.

## Introduction

It has long been recognized that diversity is richer in tropical biotas than in their temperate, high latitude counterparts [1]. However, the cause of this latitudinal diversity gradient has been the subject of much discussion (for a review see [2]). In the case of birds, this pattern is very clear in the Americas because avian diversity is particularly high in the Neotropics, which is home to 3,370 breeding landbird species (more than one third of the global avifauna), and comparatively low in the Nearctic (732 breeding species) [3]. Traditional explanations centred on the temporal stability of tropical environments, which would result in lower extinction rates and an accumulation of species [4], [5]. However, evidence of Neotropical climate and habitat change during the late Pliocene and the Pleistocene glacial cycles has led to the refuge hypothesis, which posits that the fragmentation of populations into refugia resulted in increased speciation rates during this period and the consequently high Neotropical diversity seen today [6]. This theory, which is actually only one of several explanations invoking higher speciation rates in tropical regions [2], soon became the accepted paradigm for the interpretation of avian diversity patterns, not only in the Neotropics but also in the Nearctic and other biogeographic regions. In contrast to the stability hypothesis, which predicts that species in regions with high biodiversity should be older than those in low diversity regions, the refuge hypothesis (as well as other explanations invoking higher recent speciation rates in the tropics) has the corollary that species should be younger in high diversity regions.

Debate on the timing of avian speciation and the role of Pleistocene glacial cycles has gained renewed vigour as molecular analyses have provided data on genetic divergence, enabling estimates of species ages [7]–[15] (note that criticism of the refuge hypothesis in relation to Neotropical diversification have also stemmed from biogeographical studies [16], [17] and other disciplines, such as palaeoecology [18], [19]). This debate has led to an integrated, continental-scale picture of the diversification processes when patterns were analyzed in relation to latitude [3], [20]–[25]. In the Nearctic, and particularly in northern regions, Pleistocene glacial cycles appear to have been the main force shaping current diversity, reflecting the role of ice sheets in isolating populations and often provoking vicariant speciation [3], [23]–[25]. As a consequence, many boreal species (including birds but also other groups such as mammals) show reduced genetic variation and limited phylogeographic structuring due to their recent expansion from glacial refugia [20]–[22], [24]. By contrast, Pleistocene glacial cycles had lesser impacts in the southern regions of North America and even less so in the low latitude areas of the Neotropics. Although they promoted diversification in these regions, they generated fewer speciation events than in the boreal region of the Nearctic [3], [9], [23], [25].

Reflecting this situation, it has been recently proposed that speciation rates in New World birds and mammals are lowest near the equator and increase with latitude [23], [26]. Consistent with this notion, recent studies have shown that rates of phenotypic evolution (such as avian colour patterns [27] and vocalizations [28]) are faster at higher latitudes. According to this hypothesis, the higher biodiversity in the tropics can be explained by an even steeper increase in extinction rates with latitude, which would cause a higher net rate of diversification at lower latitudes [26].

In addition to the effects of glacial cycles, or in some cases in combination with them, other geological factors are thought to have impacted avian diversity in the Neotropics. The Andes Mountains are a barrier to gene flow between populations occurring on alternate sides of the range (or between populations at high and low elevations) and also promote diversification of highland taxa [14], [17], [29]–[35]. Other factors that are thought to promote diversification include river barriers (especially in the Amazonian region [36], [37]) and marine incursions during interglacial periods in the Pliocene and Pleistocene [38], [39].

Current understanding of diversification in the Americas is based mainly on studies of North American taxa and to a lesser extent the tropical and subtropical regions of the Neotropics [40]. Much less is known about species diversification in the Southern Cone of South America (i.e. the area comprised by Argentina, Chile, Uruguay, southern Paraguay, and extreme south-eastern Brazil). This area is of particular interest because it shares certain characteristics with the Nearctic, including climate and greater exposure to glacial cycles [41], [42], but in other aspects resembles the low latitude portion of the Neotropics, including exposure to diversification agents such as the Andes Mountains and marine incursions and the presence of many lineages of birds that are shared with the whole Neotropical region. Concordantly, recent analyses performed on mammals in this region have shown a similar but more complex pattern than that in North America. High latitude populations tend to exhibit signs of recent expansions following Pleistocene glacial retreats and populations at lower latitudes appear to be more stable [43]–[45], but in a considerable proportion of Patagonian species high latitude populations show phylogeographic structuring that has originated *in situ* [44].

The Palearctic was also strongly affected by glacial cycles, but with notable differences from the Nearctic. Firstly, the ice advanced south to 40°N in North America, but did not extend past 50°N in Eurasia [3], [20]. Additionally, although there were northern, non-glaciated refuges in both regions, including Beringia [3], [24], central United States [46] and the Carpathians [47]), the advance of glacial ice sheets was more heterogeneous in the Palearctic, as evidenced by the fact that Europe and western Asia were much more affected than eastern Asia [3], [20]. Finally, fragmentation of forest habitat was far more pronounced in the Palearctic than in the Nearctic and fragments were separated by large expanses of open habitat or by interior seas [3]. This more complex recent history of the Palearctic is consistent with the finding of more variable phylogeographic patterns in Eurasian than in North American birds (e.g. [48]).

Since the inception of DNA barcoding [49], several studies have shown its effectiveness for species identification in a broad range of animal taxa, both in vertebrates [50]–[57] and invertebrates [58]–[61]. This impacted various disciplines, including ecology (e.g. studies of diet and parasitism/parasitoidism [62]–[65]), conservation [66]–[68], food security [69], [70], air safety [71]–[73] and public health [74], [75]. All of these applications of barcoding relate to species identification, but the growing datasets of standardized gene regions that result from barcode surveys, such as the cytochrome *c* oxidase I (COI) library for animals, can also be used for other kinds of analyses, including ecological and evolutionary studies [76], [77]. Notably, DNA barcode libraries can provide useful insights into differences in speciation rates and diversification patterns at a continental scale.

The novelty of this approach lies in the fact that these datasets provide information about a standardized gene fragment spanning thousands of species over broad geographic ranges. In addition, because barcoding surveys aim for comprehensive taxonomic coverage, this approach avoids biases that might arise from the selection of specific species for analysis (which sometimes depends on availability of tissue samples), the revision of the literature, or the reliance on GenBank sequences - sequences published from phylogeographic analyses might be biased towards species with high genetic variation and strong geographic structure. Moreover, because accurate estimation of species ages requires the identification of sister species, large barcoding libraries with relatively comprehensive taxon coverage facilitate the analysis of these patterns.

We have recently assembled DNA barcodes for approximately 50% of the Argentine avifauna [55], providing a dataset for the southern Neotropics that can be compared with an almost complete dataset for North American birds [54] and with a partial set (approximately 50%) of Palearctic birds [56], [57]. Here we analyze these COI datasets and compare the patterns of diversification among regions. This comparison contributes to the emerging picture of avian diversification at a global scale and also sheds light on the evolutionary patterns in the Southern Cone of South America.

## Methods

Analyses were based on a comparison of the currently available large-scale datasets of avian DNA barcodes (COI sequences), all publicly available at the Barcode of Life Data Systems (BOLD, www.boldsystems.org). These datasets correspond to the birds of: a) Argentina [55], available in the project “Birds of Argentina - Phase I” (project code: BARG, 1594 specimens, 500 species, 50% of the avifauna of Argentina, GenBank accession numbers: FJ027014 - FJ028607); b) North America [54], available in the project “Birds of North America - Phase II” (project codes: TZBNA, BNACA, BNABS and BNAUS, 2574 specimens, 643 species, 93% of the breeding and pelagic birds from the USA and Canada, GenBank accession numbers: AY666171 - AY666596, DQ432694 - DQ433261, DQ433274 - DQ433846 and DQ434243 - DQ434805), c) Eastern Palearctic [56], available in the project “Birds of the eastern Palearctic” (project code: BEPAL, 1674 specimens, 398 species, 42% of the Palearctic breeding landbird species, GenBank accession numbers: GQ481247 - GQ482920), and d) Scandinavia [57], available in the project “Birds of Scandinavia” (project codes: NORBI and SWEBI, 957 specimens, 296 species, 32% of the Palearctic breeding landbird species, GenBank accession numbers: GU571207 - GU572163). The two latter projects were merged to obtain a single dataset for the Palearctic including 2636 specimens and 488 species (52% of the breeding landbird species of the Palearctic). We therefore used three sets of COI records, which were re-analyzed to calculate standardized measurements that facilitated direct comparisons.

Some taxa that were considered single species when the aforementioned projects were originally published are now recognized as two or more species (e.g. *Cinclodes fuscus* [78]), but we have maintained the original classification used in the datasets without splitting (or lumping) species.

For all sequence comparisons, we used the Kimura 2-parameter (K2P) model [79] because it is considered to be the best metric to compare closely related taxa [80]. Neighbour-joining trees were generated using the “Taxon ID Tree” tool on BOLD.

Average intraspecific distance was calculated using all of the species in each region that were represented by at least two specimens: 389 species from Argentina (78% of the species present in the dataset), 528 species from North America (82%) and 433 species from the Palearctic (89%). The mean distance was used in each species, irrespective of the number of specimens by which it was represented. For this calculation a few low quality sequences from the North American dataset were excluded (i.e. those with more than 1% ambiguous sites) to avoid inflating distance calculations (the other two datasets did not include low quality sequences that could have this effect). We also calculated the average intraspecific distance without considering species that showed two or more lineages with high divergence (see below) because: a) some of these lineages might actually represent different species and b) a small percentage of highly variable species increases the average distance considerably and as a consequence the value obtained might not be representative of the level of variation present in most of the species of the region. ANOVAs were employed to assess whether average intraspecific distances (with and without highly variable species) differed among the three regions. In case they differed, pairs of regions were compared using post hoc Scheffé contrasts.

Species that showed two or more haplogroups in the neighbour-joining tree with a maximum intraspecific distance greater than 1.5% were considered as highly variable (this distance was chosen because all intraspecific haplogroups with bootstrap support higher than 98% were separated by at least 1.5% sequence distance in the dataset from Argentina; [55]). The proportion of highly variable species was compared among regions through a Chi-Square test, and the geographic patterns of intraspecific lineages were also analyzed and categorized as allopatric, parapatric or sympatric (in the case of the Palearctic this categorization was not possible because in the original studies [56], [57] museum samples were chosen for analysis with the goal of covering as much as possible of the distribution of each species, and therefore typically only one or two samples were included from each locality).

We used two complementary estimates for the divergence between closely related species in each region: the distance between nearest congeneric neighbours and the distance between sister species pairs. Several of the pairs of nearest congeneric neighbours in each dataset are not sister species because: a) one or both species of a pair of closest neighbours might actually be more closely related to species that are not present in the region being analyzed, and b) taxonomic coverage is not complete in two of the three datasets. As a consequence distance between nearest neighbours is expected to be higher than that between sister species (and particularly in the case of the partial datasets). Because of this, using species pairs that have been previously identified as sister species is more appropriate to compare the timing of speciation events that gave rise to extant species. Unfortunately, the datasets of the Palearctic and the southern Neotropics currently include relatively few known sister species pairs (due partly to sampling density and partly to limited phylogenetic knowledge of these avifaunas as compared to that of North American birds), thus complicating statistical analyses of resultant patterns. In addition, distances to the nearest congeneric neighbour are based solely on the result of these COI analyses, whereas the delimitation of pairs of sister species depends on previous phylogenetic and phylogeographic studies and this can bias the analysis (mainly because of the aforementioned difference in the depth in which these regions have been studied, see [81]). Because of these reasons, we consider that these analytical approaches complement each other and incorporated both of them to determine if they show similar trends.

To determine the distance between nearest congeneric neighbours, we only considered pairs of congeneric species that appeared as closest taxa in the neighbour-joining tree of each dataset (89 species pairs from Argentina, Table S1; 151 species pairs from North America, Table S2; and 93 species pairs from the Palearctic, Table S3). This procedure avoids the inclusion of any species more than once during the calculation and thus excludes non-independent comparisons, a problem faced if the distance between every species and its nearest congener is considered irrespective of their position in the tree. Species flagged as highly variable by their barcodes (see above) were excluded from the list of nearest congeneric neighbours because in some cases they might actually include two or more overlooked species.

We reviewed each pair of nearest congeners to determine whether they should be treated as sister species and only considered those that were either regarded as sister species in previous studies [12], [26], [82] or were demonstrated as such in phylogenetic or phylogeographic analyses that included all members of the genus. With this procedure we identified 13 sister species pairs from the southern Neotropics, 47 pairs from the Nearctic and 11 pairs from the Palearctic (see Tables S1, S2 and S3, respectively).

The average distances between closest congeners and between sister species were compared among regions through ANOVAs. Upon significance, pairs of regions were compared using post hoc Scheffé contrasts. The distributions of distances between nearest congeneric neighbours and between sister species pairs were compared among regions using a Kolmogorov Smirnov's two-sample Z statistic and the incidence of very recently diverged species (less than 1% sequence distance) was analyzed in each region and compared among them using a Chi-Square test.

All statistical analyses were performed using SPSS 15.0.1 and tests were two-tailed. Averages are reported with their standard deviations.

## Results

### Avian diversification in the Americas

The comparison of distances between closely related species suggested that species are older in the southern Neotropics than in the Nearctic. Average distance to the nearest congeneric neighbour was significantly higher in Argentina than in North America (6.1%±3.7% and 4.5%±3.3%, respectively; Scheffé contrast, *p* = 0.004). As expected, average distance between sister species pairs was lower than that between closest congeners, but the comparison between regions showed a similar pattern to that of the nearest neighbours; distances were higher in the southern Neotropics than in North America (4.6%±4.1% and 3.2%±2.8%, respectively). Although this pattern lacks statistical support (Scheffé contrast, *p* = 0.36), this is likely due to the small number of sister species in the dataset from the southern Neotropics.

**Figure 1:**
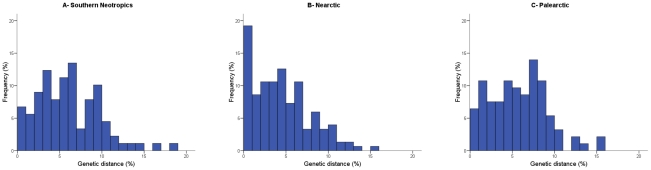
Distribution of nearest congeneric neighbour distances in COI for bird species in three biogeographic regions. Distances (calculated using the K2P model) tend to be higher in the southern Neotropics than in North America and the incidence of species pairs with very low divergence (less than 1% sequence distance) is much higher in the latter. Divergence values in the Palearctic are closer to those of the Neotropics than to those in the Nearctic.

**Figure 2:**
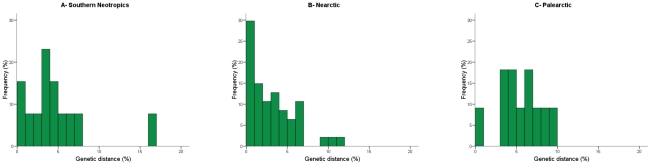
Distribution of sister species distances in COI for bird species in three biogeographic regions. Distances (calculated using the K2P model) appear to be higher in the southern Neotropics than in North America and in the later the proportion of species pairs with very low divergence (less than 1% sequence distance) is much higher. The pattern in the Palearctic is closer to that of the Neotropics than to that of the Nearctic.

The distribution of distances between nearest congeneric neighbours and between sister species can complement the overview obtained with average values. The comparison of this distribution for the nearest congeneric neighbours showed a significant difference between the southern Neotropics and the Nearctic (Figures 1A and 1B, respectively; Kolmogorov-Smirnov, Z = 1.55, *p* = 0.02). Distances tended to be lower in the Nearctic and the incidence of species pairs with very low divergence was significantly higher; only 6.7% of the nearest congeneric neighbours diverge by less than 1% sequence distance in Argentina, but this figure rises to 18.5% in North America (χ^2^ = 6.39, *p* = 0.01). When sister species were compared, the same pattern was obtained (Figures 2A and 2B); distances were lower in the Nearctic, which in particular doubled the southern Neotropics in the proportion of species with divergences lower than 1% (15.4% in the Neotropics vs. 29.8% in the Nearctic). Although neither difference was statistically significant (difference in the distribution, Z = 0.825, *p* = 0.50; proportion of species with less than 1% divergence, χ^2^ = 1.06 *p* = 0.30), this is likely due to the small number of sister species in the Argentine dataset. It should be mentioned that the higher proportion of species with very low divergence in the Nearctic could partially reflect the more intense taxonomic scrutiny of birds in this region than in the Neotropics. This could lead to more cases of narrowly divergent taxa being elevated to full species in the Nearctic. However, because the distribution of distances as a whole was significantly different between these regions, with a clear tendency of Nearctic species pairs to be less diverged (see Figures 1A, 1B, 2A and 2B), this taxonomic bias appears not to be the reason for this pattern.

The difference in the patterns of divergence between pairs of closely related species reinforces the conclusion that Neotropical species are older than their Nearctic counterparts. This difference also explains why DNA barcodes had higher success in species identification in the southern Neotropics than in the Nearctic (98% and 94%, respectively), because all pairs or groups of species that could not be separated through COI in North America diverged by less than 1%. Critics of DNA barcoding argued that the Neotropics would pose a challenge because of high species richness and high intraspecific divergence [83]. Our results suggest that this is not the case. In fact, unless interspecific divergence was lower in the Neotropics there would be no reason for barcoding to fail more than in other regions. Higher intraspecific variation would lead to an increase of species flagged by barcoding as interesting for deeper analysis and hence augment its usefulness as a tool for species discovery, but would not compromise its efficiency for species identification.

Average intraspecific distances were 0.24%±0.41% in the southern Neotropics and 0.28%±0.46% in the Nearctic (Scheffé contrast, *p* = 0.43), suggesting that the avifaunas in these two regions have similar (and highly variable) levels of intraspecific variation. Apart from comparing these averages, the analysis of species with high variation in each region could advance the understanding of the processes that generate diversification in the Americas. In our study of Argentina, we analyzed in detail those species that showed two or more deeply divergent haplogroups in the neighbour joining tree (diverging by at least 1.5% sequence distance and having a bootstrap support higher than 98% in all cases). In total, 5.4% of the species that were represented by multiple individuals fell on this category (21 out of 389, see table 1). In the Nearctic dataset, 4.0% of the species were included in this group of highly variable taxa (21 out of 528, see Table 2). This shows that the proportion of species with geographic structure and deeply divergent intraspecific lineages is similar in the two regions (the difference is not significant, χ^2^ = 1.04, *p* = 0.31).

**Table 1:** Avian species from Argentina showing two or more deeply divergent haplogroups at COI and intraspecific variation higher than 1.5%.

Family	Species	Max. intraspecific distance (%)*
Charadriidae	*Vanellus chilensis*	1.54
Strigidae	*Athene cunicularia*	1.60
Dendrocolaptidae	*Sittasomus griseicapillus*	3.25
Furnariidae	*Geositta cunicularia*	3.41
	*Cinclodes fuscus*	4.65
	*Leptasthenura aegithaloides*	3.72
	*Upucerthia dumetaria*	5.41
	*Cranioleuca pyrrhophya*	1.53
Thamnophilidae	*Thamnophilus ruficapillus*	4.03
	*Thamnophilus caerulescens*	2.44
Tyrannidae	*Serpophaga subcristata*	2.04
	*Myiophobus fasciatus*	4.67
	*Knipolegus aterrimus*	1.9
Pipridae	*Manacus manacus*	3.56
Vireonidae	*Vireo olivaceus*	3.09
Troglodytidae	*Cistothorus platensis*	4.95
	*Troglodytes aedon*	4.99
Thraupidae	*Thraupis bonariensis*	3.29
	*Saltator aurantiirostris*	1.52
Emberizidae	*Arremon flavirostris*	1.75
Cardinalidae	*Cyanocompsa brissonii*	2.04

*Distances were calculated using the K2P model (see Methods section).

**Table 2:** Avian species from the Nearctic showing two or more deeply divergent haplogroups at COI and intraspecific variation higher than 1.5%.

Family	Species	Max. intraspecific distance (%)*
Anatidae	*Branta canadensis*	1.8
	*Bucephala islandica*	1.6
Phasianidae	*Lagopus lagopus*	2.2
Procelariidae	*Fulmarus glacialis*	3.1
Scolopacidae	*Tringa solitaria*	5.4
Strigidae	*Megascops kennicottii*	3.1
Tyrannidae	*Contopus sordidulus*	1.6
Vireonidae	*Vireo gilvus*	4.0
Sittidae	*Sitta canadensis*	2.2
Corvidae	*Aphelocoma californica*	5.3
	*Aphelocoma ultramarina*	3.2
	*Corvus corax*	4.3
Paridae	*Poecile gambeli*	3.7
Aegithalidae	*Psaltriparus minimus*	3.6
Troglodytidae	*Thryomanes bewickii*	4.8
	*Troglodytes troglodytes*	6.4
	*Cistothorus palustris*	7.9
Turdidae	*Catharus guttatus*	3.2
Mimidae	*Toxostoma curvirostre*	7.4
Emberizidae	*Chondestes grammacus*	1.6
Icteridae	*Sturnella magna*	4.6

*Distances were calculated using the K2P model (see Methods section).

However, the patterns of diversification observed were very different in the two regions. In North America, most cases of deep intraspecific variation involved allopatric divergence between eastern and western populations, a pattern suggesting divergence in glacial refugia along the margins of the continent. The patterns in Argentina were more complex. Species with allopatric, parapatric and sympatric lineages were found and geographic patterns were diverse, including cases of eastern and western lineage differentiation, northern and southern divergence and even apparent isolation among populations at varying elevations in the Andes Mountains.

We expect that many of these divergent lineages actually constitute different species. Splits had been recommended for some of these species based on sequence analysis at other gene loci, morphology or behaviour before barcoding studies were performed, but they were treated as conspecific due to a lack of consensus on their elevation to full species (these cases and the corresponding citations are mentioned in the original articles; [54], [55]). In other cases, subsequent studies confirmed that divergent lineages warrant species status (e.g. [78]). In this sense, the diversification patterns described above might in some cases include closely related species (usually sister species) and not intraspecific lineages, a problematic distinction often faced by studies analyzing recent speciation events [9], [13]. Although this complexity does not alter conclusions about diversification patterns or processes in each region, it might be more appropriate to compare average intraspecific variation without considering these highly variable taxa. In addition, both the average intraspecific distance and its variance increase considerably when this relatively small percentage of variable species is included in the calculation and might not represent the level of intraspecific variation present in most of the species of each region. These recalculations showed that when highly variable species are excluded, intraspecific distance is still slightly higher in the Nearctic (0.21%±0.23%) than in the southern Neotropics (0.16%±0.19%), but in this case the difference is significant (Scheffé contrast, *p* = 0.003).

### Comparing the Americas and the Palearctic

In the avifauna from the Palearctic average distance to the nearest congeneric neighbour was 5.8%±3.4%. This value is more similar to that for Argentina (6.1%) than for North America (4.4%), which was confirmed by the lack of significance in the comparison with the southern Neotropics (Scheffé contrast, *p* = 0.86) and a significant difference in the comparison with the Nearctic (*p* = 0.02). Consistently, the distribution of distances to the nearest congeneric neighbour (Figure 1C) was similar and not significantly different between the Palearctic and the southern Neotropics (Z = 0.90, *p* = 0.39) but significantly different between the Palearctic and the Nearctic (Z = 1.61, *p* = 0.01). Divergences in Eurasia tended to be relatively high and, in particular, 6.5% of nearest congeneric neighbours diverged by less than 1% sequence distance, a percentage almost identical to that obtained for Argentina (6.7%; χ^2^ = 0.006, *p* = 0.94) but significantly lower than the percentage observed in North America (18.5%; χ^2^ = 6.99, *p* = 0.008).

The comparison based on pairs of sister species gave almost identical results. The average distance between sister species in the Palearctic was 5.6%±2.5%, a value closer to that for the southern Neotropics (4.6%) than for the Nearctic (3.2%). In fact, the difference with the southern Neotropics was not significant (Scheffé contrast, *p* = 0.73) but the difference with North America was marginally significant (p = 0.07). The same trend was observed when comparing the distribution of distances; no differences were detected between the Palearctic and the southern Neotropics (Z = 0.91, *p* = 0.39), but a significant difference was found between the Palearctic and North America (Z = 1.61, *p* = 0.01). Regarding pairs of sister species with very low levels of divergence, 9.1% of sister species had less than 1% sequence distance in the Palearctic, a value slightly lower than that of the southern Neotropics (15.4%) but markedly lower than that of the Nearctic (29.8%). Likely due to the fact that only 11 sister species pairs could be confirmed in the Palearctic dataset, none of these differences were statistically significant (comparison with the Neotropics, χ^2^ = 0.22, *p* = 0.64; comparison with the Nearctic, χ^2^ = 1.99, *p* = 0.16).

Average intraspecific distance of the Palearctic dataset was 0.32%±0.53%. This value is similar to that found for the Nearctic (0.28%; Scheffé contrast, *p* = 0.37) but higher than that for the southern Neotropics (0.24%; Scheffé contrast, *p* = 0.04). However, if one excludes the highly variable species (which represent 10% of the total, see below) this figure drops to 0.19%±0.18%, a value that falls between the equivalent measurement for the southern Neotropics (0.16%) and North America (0.21%) and does not differ significantly from either of them (Scheffé contrasts, *p* = 0.31 and *p* = 0.18, respectively). This result indicates that the higher average intraspecific distance obtained initially in this region was inflated by the presence of a higher percentage of species with divergent lineages in comparison with the other two regions.

As mentioned above, 10% of the species from the Palearctic represented by multiple individuals had at least two haplogroups with over 1.5% maximum distance (43 out of 433 species, see Table 3). This percentage is significantly higher than those of variable species in the southern Neotropics (5.4%; χ^2^ = 5.86, *p* = 0.02) or the Nearctic (4%; χ^2^ = 13.56, *p* = 0.002). Despite this higher percentage of variable species, the geographic patterns showed by their divergent lineages are not particularly complex. In fact, complexity appeared intermediate between North America and Argentina, showing three main patterns: a) east-west differentiation, b) one lineage dispersed through most of the Palearctic and the other one restricted to the Sakhalin region, and c) one lineage dispersed through most of the Palearctic and the other one restricted to the Caucasus region (in most cases the northeastern coast of the Black Sea).

**Table 3:** Avian species from the Palearctic showing two or more deeply divergent haplogroups at COI and intraspecific variation higher than 1.5%.

Family	Species	Max. intraspecific distance (%)*
Phasianidae	*Perdix perdix*	1.7
	*Phasianus colchicus*	2.3
Rallidae	*Rallus aquaticus*	3.2
Scolopacidae	*Limosa limosa*	2.4
Alcidae	*Uria lomvia*	1.9
Columbidae	*Streptopelia orientalis*	2.4
Cuculidae	*Cuculus canorus*	3.2
Caprimulgidae	*Caprimulgus europaeus*	3.0
Picidae	*Dendrocopos major*	2.8
	*Dendrocopos minor*	1.6
Laniidae	*Lanius collurio*	2.8
	*Lanius escubitor*	3.9
Alaudidae	*Alauda arvensis*	8.7
Hirundinidae	*Delichon dasypus*	6.4
Corvidae	*Perisoreus infaustus*	2.1
	*Garrulus glandarius*	2.9
	*Pica pica*	3.9
	*Corvus frugilegus*	3.1
	*Corvus corone*	2.5
Paridae	*Periparus ater*	4.7
	*Parus major*	3.1
Cettidae	*Urosphena squameiceps*	2.2
	*Cettia diphone*	3.3
Siitidae	*Sitta europaea*	12.2
Certhiidae	*Certhia familiaris*	2.2
Troglodytidae	*Troglodytes troglodytes*	3.9
Regulidae	*Regulus regulus*	3.9
Phylloscopidae	*Phylloscopus borealis*	3.9
	*Phylloscopus trochiloides*	5.1
Silviidae	*Sylvia curruca*	5.8
Muscicapidae	*Muscicapa sibirica*	2.9
	*Erithacus rubecula*	5.0
	*Luscinia megarhynchos*	2.7
	*Phoenicurus ochruros*	4.5
	*Phoenicurus phoenicurus*	5.4
	*Phoenicurus auroreus*	2.4
	*Saxicola maurus*	8.1
Sturnidae	*Sturnus vulgaris*	2.0
Motacillidae	*Motacilla flava*	6.0
Emberizidae	*Emberiza pusilla*	2.0
Emberizidae	*Emberiza spodocephala*	3.6
Emberizidae	*Emberiza pallasi*	3.2
Fringillidae	*Uragus sibiricus*	1.8

*Distances were calculated using the K2P model (see Methods section).

## Discussion

We used currently available large-scale datasets of avian COI sequences to compare diversification patterns at a continental scale in different biogeographic regions, an endeavour which extends the use of barcodes beyond species identification and taxonomy.

Our analysis suggests that avian species are older in the southern Neotropics than in the Nearctic, as evidenced by average genetic distances between pairs of closely related species, the distribution of these distances, and the proportion of very recently diverged pairs of species - a trend shown both by nearest congeneric neighbours and sister species. This result supports the conclusion that Pleistocene glacial cycles had more pronounced effects in the Nearctic (particularly in boreal North America) than in the Neotropics, isolating populations and promoting vicariant speciation [3], [20]-[25]. In turn, this result also supports analyses that indicate that recent speciation rates are lower in the Neotropics than in the Nearctic [23], [26], a pattern opposite to that expected under the prevailing view of higher recent speciation rates at low latitudes [2] and to the predictions of the refuge paradigm. The finding of similar patterns in the Palearctic and the Southern Cone of South America is consistent with a milder effect of Pleistocene glaciations in the Palearctic than in the Nearctic.

Apart from allowing relative comparisons of the age of species among regions, the divergence values of COI between sister species can be used to estimate absolute species ages. However, this requires knowledge of the evolutionary rate of COI and formal calibrations of this gene have not yet been performed. Calibrations of avian mitochondrial DNA have relied mainly on cytochrome *b* [84], [85], for which a molecular rate of approximately 2.1% per million years has been shown to be relatively conserved [85]. COI has been suggested to evolve more slowly than cytochrome *b* in birds [86] and there is also evidence for variation in its molecular rate across taxa [82], [87]. We performed a calibration of COI relative to cytochrome *b* for the pairs of sister species included in our datasets by downloading cytochrome *b* sequences from GenBank. There were sequences for both members of a species pair for 51 of the 71 pairs identified in our study (33 from the Nearctic, 10 from the Palearctic, and 7 from Argentina). Divergence in cytochrome *b* was determined between each sister species pair (using the K2P model) and compared to our previous results for COI. This estimation indicated that COI appears to evolve 35% slower than cytochrome *b* in this set of sister species, a result almost identical to that of Aliabadian et al. [86] for intrageneric comparisons. If the evolutionary rate of 2.1% per million years of cytochrome *b* is considered, this result indicates that COI molecular rate is about 1.4% per million years.

Because sequence distance in about 30% of sister species pairs in the dataset from the Nearctic is less than 1%, the application of the molecular clock discussed above implies that speciation events that led to almost one third of avian sister species pairs in this region occurred in the last 700,000 years (mid to late Pleistocene). By contrast, considerably fewer southern Neotropic and Palearctic sister species pairs appear to have diverged so recently (about 15% and 10%, respectively). Only 13 and 11 sister species pairs were considered in the datasets for the Neotropics and the Palearctic, respectively, and likely as a consequence of this the differences between regions were not statistically significant (only the difference between the Palearctic and the Nearctic was marginally significant). However, the comparison of nearest congeneric neighbours, which included many more pairs of species from these regions (89 in the case of the southern Neotropics and 93 in the case of the Palearctic), also showed nearly three times as many species pairs with less than 1% divergence in the Nearctic than in the other two regions and the differences were statistically significant. It should be borne in mind that even though nearest congeneric distances could decrease as the datasets from the southern Neotropics and the Palearctic continue to be populated, this effect is not likely to be very pronounced. This is because some groups or clades in these datasets are fairly well represented while others are missing almost entirely; thus, fewer pairs of species are erroneously considered as nearest neighbours than would be if unsampled species were evenly distributed among groups or clades.

At the other extreme, almost half of sister species pairs (22 of 47) diverge by more than 2.8% in the Nearctic, implying that the origin of about half of extant Nearctic sister species could be traced to the Pliocene. In the southern Neotropics, however, almost 70% of sister species appear to have originated in the Pliocene (9 of 13 sister species pairs, including the case of the pair *Tinamotis ingoufi* - *T. pentlandii,* which diverge by more than 16% and thus probably split before the Pliocene) and more than 90% in the Palearctic (10 of 11 sister species). This shows that not only the percentage of species that have originated very recently is higher in the Nearctic, but that the proportion of species originated throughout the entire Pleistocene appears to be higher in this region.

Levels of intraspecific diversification appear similar in the Nearctic and the southern Neotropics, as reflected both in the average intraspecific distance and the proportion of species with high variation. This result was unexpected because it is commonly assumed that a much higher percentage of species in the Neotropics are variable or possess geographic structuring than in North America. In the Palearctic, average intraspecific variation considering all species was higher than in the New World. However, when the relatively high proportion of highly variable species in this region was excluded, intraspecific variation was similar to that of the Americas. The higher proportion of variable species in the Palearctic could partially reflect its much larger area (about 46 million km^2^; [3]) compared to that of the Nearctic (18.2 million km^2^; [3]) and Argentina (about 3 million km^2^) or the inclusion of a higher proportion of the range of the species of this dataset in comparison with the Americas (particularly in the southern Neotropics, where Argentina represents only a relatively small portion of the distribution range of many species). Nevertheless, the heterogeneous advance of the ice in the Palearctic during the Pleistocene [3], [20], the variable topography of southern Europe [24], [88] and the pronounced fragmentation of forests in Eurasia during glacial maxima [3] could also explain the presence of geographic structure in a higher proportion of Palearctic bird species.

The detailed analysis of geographic patterns of lineages in species with high variation showed marked differences in complexity among regions. In the Nearctic, almost all variable species showed eastern and western lineages, a pattern already reported in birds [23] and mammals [46], [89], [90] that is consistent with population expansions from glacial refugia in the margins of North America. The eastern and western portions of the Nearctic probably acted as refugia during the early and mid Pleistocene, but were later covered by ice [23]. This is consistent with the fact that the eastern and western lineages within species typically show more than 1.5% divergence at COI. In the Palearctic three patterns were found, all previously reported in birds and other groups of animals, and interpreted at least partially as evidence of range expansion from refugia after the retreat of the ice or permafrost that covered much of the northern portion of Eurasia [24], [48], [88], [91]. Finally, in the Neotropics several patterns were found and they appear to reflect various sources of diversification, including the Andes Mountains. These differences once again support the conclusion of a major effect of Pleistocene glaciations in the Nearctic, a less marked and more heterogeneous effect in the Palearctic and an even milder effect in the southern Neotropics, where other diversification processes may have been as relevant in shaping current diversity.

Focusing on the New World, in spite of the more complex geographic patterns of divergent intraspecific lineages in the southern Neotropics versus the Nearctic, both regions showed similar levels of intraspecific variation. Considered jointly, these results suggest that even though diversification processes are more complex in the southern Neotropics, this difference does not generate diversification in a much higher percentage of species. This is not only unexpected because of the aforementioned tendency to assume that there are more variable species in the Neotropics [21], [22], but also because of the diversity of factors that appear involved as diversifying agents (including the Andes Mountains [14], [17], [29]–[35], large rivers [36], [37] and marine incursions [38], [39]), some of which were in fact reflected in the various patterns of geographic distribution of lineages found.

In summary, the comparative analysis of the COI dataset for the birds of Argentina (the first large scale dataset of barcodes of Neotropical vertebrates) with those for the Nearctic and Palearctic has provided information about avian patterns of diversification at a continental scale, including information on the understudied Southern Cone of South America. Once barcoding projects generate large-scale datasets in more tropical areas of the continent it will be possible to evaluate whether the patterns found are representative of the whole Neotropical region. The recent establishment of large-scale barcoding projects in other South American countries (such as Bolivia) will contribute with this objective. In fact, preliminary analysis of the Argentine dataset in combination with recent results from Bolivia detected a higher proportion of bird species with deeply divergent lineages (unpublished data), a pattern that could be related to both the inclusion of more tropical areas and to better coverage of species' ranges. The assessment of this emerging trend will not have to wait for long if barcoding surveys continue at the current pace.

## Supporting Information

Table S1 **List of nearest congeneric neighbours in the dataset from Argentina.** Taxonomic information for each pair and its genetic distance (K2P) are provided. Pairs identified as sister species are in bold and the references used to identify them are listed.
(DOC)

Table S2 **List of nearest congeneric neighbours in the dataset from the Nearctic.** Taxonomic information for each pair and its genetic distance (K2P) are provided. Pairs identified as sister species are in bold and the references used to identify them are listed.
(DOC)

Table S3 **List of nearest congeneric neighbours in the dataset from the Palearctic.** Taxonomic information for each pair and its genetic distance (K2P) are provided. Pairs identified as sister species are in bold and the references used to identify them are listed.
(DOC)
