# Supplementary material for: DNA Barcode Libraries Provide Insight into Continental Patterns of Avian Diversification
Source: PLoS One. 2011 Jul 27;6(7):e20744. doi: 10.1371/journal.pone.0020744 (PMC3144888; doi:10.1371/journal.pone.0020744)
Supplement: Table S1 — List of nearest congeneric neighbours in the dataset from Argentina. Taxonomic information for each pair and its genetic distance (K2P) are provided. Pairs identified as sister species are in bold and the references used to identify them are listed. (DOC) [file pone.0020744.s001.doc]

**Table S1**. List of nearest congeneric neighbours in the dataset from Argentina. Taxonomic information for each pair and its COI genetic distance (K2P) are provided. Pairs identified as sister species are in bold and the references used to identify them are listed.

| **Oder** | **Family** | **Species pair** | **COI genetic distance** | **Reference** |
| --- | --- | --- | --- | --- |
| Tinamiformes | Tinamidae | *Nothura darwinii - N. maculosa* | 9.395 |  |
|  |  | ***Tinamotis ingoufi - T. pentlandii*** | **16.484** | 1 |
| Anseriformes | Anatidae | *Dendrocygna autumnalis - D. viduata* | 14.042 |  |
|  |  | *Chloephaga picta - C. poliocephala* | 5.221 |  |
|  |  | *Anas cyanoptera - A. platalea* | 2.751 |  |
|  |  | *Anas bahamensis - A. georgica* | 2.639 |  |
|  |  | *Anas puna - A. versicolor* | 0.676 |  |
| Phoenicopteriformes | Phoenicopteridae | *Phoenicopterus andinus - P. jamesi* | 1.757 |  |
| Procellariiformes | Pelecanoididae | ***Pelecanoides georgicus - P. magellani*** | **6.912** | 2 |
| Pelecaniformes | Phalacrocoracidae | *Phalacrocorax atriceps - P. brasilianus* | 6.441 |  |
| Ciconiiformes | Ardeidae | *Egretta caerulea - E. thula* | 8.055 |  |
| Falconiformes | Accipitridae | *Ictinia mississippiensis - I. plumbea* | 4.598 |  |
|  |  | *Circus buffoni - C. cinereus* | 7.979 |  |
|  |  | *Buteogallus meridionalis - B. urubitinga* | 6.273 |  |
|  | Falconidae | *Falco peregrinus - F. sparverius* | 9.683 |  |
| Gruiformes | Rallidae | *Aramides cajanea - A. ypecaha* | 4.376 |  |
|  |  | *Fulica armillata - F. gigantea* | 2.394 |  |
| Charadriiformes | Charadriidae | *Charadrius alticola - C. falklandicus* | 1.507 |  |
|  | Haematopodidae | *Haematopus ater - H. leucopodus* | 3.968 |  |
|  | Scolopacidae | *Tringa flavipes - T. melanoleuca* | 9.985 |  |
|  |  | *Calidris bairdii - C. melanotos* | 9.285 |  |
|  | Thinocoridae | ***Attagis gayi - A. malouinus*** | **4.354** | 3 |
|  | Laridae | *Larus dominicanus - L. scoresbii* | 5.429 |  |
|  |  | *Thalasseus maximus - T. sandvicensis* | 3.394 |  |
| Columbiformes | Columbidae | *Patagioenas cayennensis - P. picazuro* | 3.031 |  |
|  |  | *Columbina picui - C. talpacoti* | 8.235 |  |
|  |  | *Metriopelia aymara - M. melanoptera* | 9.888 |  |
|  |  | *Leptotila megalura - L. rufaxilla* | 1.476 |  |
| Psittaciformes | Psittacidae | *Psilopsiagon aurifrons - P. aymara* | 6.343 |  |
|  |  | *Aratinga leucophthalma - A. mitrata* | 6.287 |  |
|  |  | *Amazona aestiva - A. tucumana* | 6.542 |  |
| Strigiformes | Strigidae | *Megascops choliba - M. hoyi* | 10.606 |  |
|  |  | *Glaucidium brasilianum - G. nanum* | 2.289 |  |
| Trogoniformes | Trogonidae | *Trogon rufus - T. surrucura* | 18.967 |  |
| Coraciiformes | Alcedinidae | *Chloroceryle amazona - C. americana* | 11.066 |  |
| Piciformes | Ramphastidae | *Ramphastos dicolorus - R. toco* | 9.043 |  |
|  | Picidae | *Picumnus cirratus - P. temminckii* | 2.202 |  |
|  |  | *Melanerpes cactorum - M. candidus* | 10.573 |  |
|  |  | *Picoides lignarius - P. mixtus* | 2.071 |  |
|  |  | ***Veniliornis frontalis - V. passerinus*** | **0.337** | 4 |
|  |  | ***Colaptes pitius - C. rupicola*** | **3.828** | 5 |
|  |  | *Campephilus leucopogon - C. magellanicus* | 5.875 |  |
| Passeriformes | Furnariidae | *Cinclodes atacamensis - C. patagonicus* | 5.338 |  |
|  |  | *Synallaxis albescens - S. azarae* | 2.658 |  |
|  |  | *Asthenes anthoides - A. modesta* | 4.153 |  |
|  |  | ***Asthenes baeri - A. dorbignyi*** | **1.461** | 6 |
|  |  | *Asthenes patagonica - A. steinbachi* | 4.817 |  |
|  |  | *Phacellodomus rufifrons - P. striaceps* | 4.672 |  |
|  |  | *Phacellodomus ruber - P. striaticollis* | 3.341 |  |
|  |  | ***Xiphocolaptes albicollis - X. major*** | **3.096** | 7 |
|  |  | *Lepidocolaptes angustirostris - L. falcinellus* | 5.744 |  |
|  | Rhinocryptidae | *Scytalopus magellanicus - S. superciliaris* | 9.494 |  |
|  | Tyrannidae | *Anairetes flavirostris - A. parulus* | 6.374 |  |
|  |  | *Pseudocolopteryx flaviventris - P. sclateri* | 6.268 |  |
|  |  | *Elaenia albiceps - E. obscura* | 12.176 |  |
|  |  | *Elaenia parvirostris - E. spectabilis* | 10.42 |  |
|  |  | *Hemitriccus diops - H. margaritaceiventer* | 11.238 |  |
|  |  | *Lessonia oreas - L. rufa* | 3.349 |  |
|  |  | *Muscisaxicola cinereus - rufivertex* | 0.507 |  |
|  |  | ***Muscisaxicola capistratus - M. frontalis*** | **0.582** | 8 |
|  |  | *Agriornis micropterus - A. murinus* | 8.627 |  |
|  |  | *Myiarchus swainsoni - M. tuberculifer* | 3.739 |  |
|  |  | *Tyrannus melancholicus - T. savana* | 1.655 |  |
|  | Cotingidae | *Phytotoma rara - P. rutila* | 10.128 |  |
|  | Tityridae | *Pachyramphus validus - P. viridis* | 13.27 |  |
|  | Corvidae | *Cyanocorax chrysops - C. cyanomelas* | 9.138 |  |
|  | Hirundinidae | ***Tachycineta leucorrhoa - T. meyeni*** | **3.333** | 9 |
|  | Turdidae | *Catharus dryas - C. ustulatus* | 8.427 |  |
|  |  | *Turdus chiguanco - T. nigriceps* | 3.483 |  |
|  | Mimidae | *Mimus dorsalis - M. triurus* | 0.888 |  |
|  | Motacillidae | *Anthus correndera - A. hellmayri* | 6.313 |  |
|  | Parulidae | *Basileuterus leucoblepharus - B. signatus* | 7.929 |  |
|  | Thraupidae | ***Tachyphonus coronatus - T. rufus*** | **5.284** | 10 |
|  |  | *Saltator coerulescens - S. similis* | 6.658 |  |
|  | Emberizidae | *Phrygilus atriceps - P. gayi* | 3.323 |  |
|  |  | ***Phrygilus plebejus - P. unicolor*** | **4.365** | 11 |
|  |  | ***Phrygilus alaudinus - P. carbonarius*** | **7.362** | 11 |
|  |  | *Poospiza erythrophrys - P. torquata* | 5.709 |  |
|  |  | *Poospiza nigrorufa - P. ornata* | 6.749 |  |
|  |  | *Sporophila caerulescens - S. collaris* | 8.417 |  |
|  |  | *Sicalis luteocephala - S. olivascens* | 8.733 |  |
|  |  | *Sicalis flaveola - S. luteola* | 9.394 |  |
|  |  | *Emberizoides herbicola - E. ypiranganus* | 5.084 |  |
|  |  | *Paroaria capitata - P. coronata* | 5.124 |  |
|  | Icteridae | *Sturnella loyca - S. superciliaris* | 5.73 |  |
|  |  | ***Pseudoleistes guirahuro - P. virescens*** | **2.131** | 4 |
|  |  | *Molothrus bonariensis - M. rufoaxillaris* | 6.677 |  |
|  | Fringillidae | *Euphonia chlorotica - E. pectoralis* | 8.899 |  |
|  |  | *Carduelis atrata - C. crassirostris* | 0.591 |  |

1. Bertelli S, Porzecanski AL (2004) Tinamou (Tinamidae) systematics:a preliminary combined analysis of morphology and molecules. Ornitología Neotropical 15: 1-7.
2. Kennedy M, Page RDM (2002) Seabird supertrees: combining partial estimates of Precellariiform phylogeny. Auk 119: 88-108.
3. Thomas GH, Wills MA, Székely (2004) A supertree approach to shorebird phylogeny. BMC Evolutionary Biology 4: 28.
4. Weir JT, Schluter D (2007) The latitudinal gradient in recent speciation and extinction rates of birds and mammals. Science 315: 1574-1576.
5. Moore WS, Overton LC, Miglia KJ (2010) Mitochondrial DNA based phylogeny of the woodpecker genera *Colaptes* and *Piculus*, and implications for the history of woodpecker diversification in South America. Molecular Phylogenetics and Evolution. In press.
6. Derryberry E, Claramunt S, O´Quin KE, Aleixo A, Chesser T, et al. (2010) *Pseudoasthenes*, a new genus of ovenbird (Aves: Passeriformes: Furnariidae). Zootaxa 2416: 61-68.
7. Raikow RJ. (1994) A phylogeny of the Woodcreepers (Dendrocolaptinae). Auk 111: 104-114.
8. Chesser RT. (2000) Evolution in the High Andes: the phylogenetics of Muscisaxicola Ground-Tyrants. Molecular Phylogenetics and Evolution 15: 369-380.
9. Whittingham LA, Slikas B, Winkler DW, Sheldon FH (2002) Phylogeny of the Swallow genus, Tachycineta (Aves: Hirundinidae), by Bayesian analysis of mitochondrial DNA sequences. Molecular Phylogenetics and Evolution 22: 430-441.
10. Burns KJ, Racicot RA (2009) Molecular phylogenetics of a clade of lowland tanagers: implications for avian participation in the great American interchange. Auk 126: 635-648.
11. Campagna L. Unpublished data.
