# Supplementary material for: DNA Barcode Libraries Provide Insight into Continental Patterns of Avian Diversification
Source: PLoS One. 2011 Jul 27;6(7):e20744. doi: 10.1371/journal.pone.0020744 (PMC3144888; doi:10.1371/journal.pone.0020744)
Supplement: Table S2 — List of nearest congeneric neighbours in the dataset from the Nearctic. Taxonomic information for each pair and its genetic distance (K2P) are provided. Pairs identified as sister species are in bold and the references used to identify them are listed. (DOC) [file pone.0020744.s002.doc]

**Table S2**. List of nearest congeneric neighbours in the dataset from the Nearctic. Taxonomic information for each pair and its COI genetic distance (K2P) are provided. Pairs identified as sister species are in bold and the references used to identify them are listed.

| **Order** | **Family** | **Species pair** | **COI genetic distance** | **Reference** |
| --- | --- | --- | --- | --- |
| Anseriformes | Anatidae | ***Chen caerulescens - C. rossi*** | **0.307** | 1 |
|  |  | *Branta leucopsis - B. hutchinsii* | 0.71 |  |
|  |  | *Cygnus buccinator - C. columbianus* | 1.72 |  |
|  |  | *Anas americana - A. strepera* | 3.016 |  |
|  |  | *Anas discors - A. cyanoptera* | 0.088 |  |
|  |  | *Aythya americana - A. collaris* | 0.907 |  |
|  |  | *Aythya affinis - A. marila* | 0.183 |  |
|  |  | *Somateria mollissima - S. spectabilis* | 0.534 |  |
|  |  | *Melanitta fusca - M. perspicillata* | 4.629 |  |
| Galliformes | Odontophoridae | ***Callipepla califronica - C. gambelli*** | **2.735** | 2 |
|  | Phasianidae | ***Centrocercus minimus - C. urophasianus*** | **0.852** | 1,3 |
|  |  | ***Dendragapus fuliginosus - D. obscurus*** | **1.257** | 3 |
|  |  | *Tympanuchus cupido - T. phasianellus* | 0.677 |  |
| Gaviiformes | Gaviidae | *Gavia arctica - G. pacifica* | 4.545 |  |
|  |  | *Gavia adamsii - G. immer* | 0.773 |  |
| Podicipediformes | Podicipedidae | *Podiceps auritus- P. grisegena* | 6.928 |  |
|  |  | *Aechmophorus clarkii - A. occidentalis* | 0.282 |  |
| Procellariiformes | Diomedeidae | ***Phoebastria immutabilis - P. nigripes*** | **0.865** | 4 |
| Procellariiformes | Procellariidae | *Puffinus carneipes - P. creatopus* | 0.187 |  |
|  |  | ***Puffinus bulleri - P. pacificus*** | **3.803** | 3,4,5 |
|  |  | *Puffinus gravis - P. tenuirostris* | 2.536 |  |
|  |  | *Puffinus lherminieri - P. puffinus* | 3.121 |  |
|  | Hydrobatidae | *Oceanodroma furcata - O. castro* | 12.653 |  |
|  |  | *Oceanodroma homochroa - O. leucorhoa* | 9.49 |  |
| Pelecaniformes | Pelecanidae | *Pelecanus erythrorhynchos - P. occidentalis* | 6.27 |  |
|  | Phalacrocoracidae | *Phalacrocorax auritus - P. brasilianus* | 3.054 |  |
|  |  | *Phalacrocorax pelagicus - P. urile* | 0.81 |  |
| Ciconiiformes | Ardeidae | *Egretta rufescens - E. tricolor* | 4.492 |  |
|  | Threskiornithidae | *Plegadis chihi - P. falcinellus* | 0.897 |  |
| Falconiformes | Accipitridae | *Accipiter cooperii - A. gentilis* | 7.689 |  |
|  |  | *Buteo brachyurus - B. swainsoni* | 1.046 |  |
|  |  | *Buteo lagopus - B. albonotatus* | 2.028 |  |
|  | Falconidae | *Falco peregrinus - F. rusticolus* | 4.427 |  |
| Gruiformes | Rallidae | ***Rallus elegans - R. longisrostris*** | **0.156** | 1 |
|  | Gruidae | *Grus canadensis - G. americana* | 4.714 |  |
| Charadriiformes | Charadriidae | ***Pluvialis dominica - P. fulva*** | **4.878** | 6 |
|  |  | *Charadrius leschenaultii - C. wilsonia* | 8.119 |  |
|  |  | *Charadrius hiaticula - C. melodus* | 8.124 |  |
|  |  | *Charadrius montanus - C. alexandrinus* | 10.751 |  |
|  | Haematopodidae | *Haematopus palliatus - H. bachmani* | 0.663 |  |
|  | Scolopacidae | *Tringa melanoleuca - T. semipalmata* | 8.951 |  |
|  |  | *Tringa flavipes - T. glareola* | 8.869 |  |
|  |  | *Numenius phaeopus - N. tahitiensis* | 6.713 |  |
|  |  | *Limosa haemastica - L. fedoa* | 6.152 |  |
|  |  | ***Arenaria melanocephala - A. interpres*** | **6.37** | 6 |
|  |  | *Calidris alba - C. fuscicollis* | 8.491 |  |
|  |  | *Calidris mauri - C. pusilla* | 4.311 |  |
|  |  | *Calidris maritima - C. ptilocnemis* | 1.4 |  |
|  |  | *Calidris himantopus - C. minutilla* | 7.491 |  |
|  |  | ***Limnodromus scolopaceus - L. griseus*** | **9.586** | 1,3,6 |
|  |  | ***Phalaropus fulicarius - P. lobatus*** | **6.085** | 3 |
|  | Laridae | ***Rissa brevirostris - R. tridactyla*** | **5.036** | 3 |
|  |  | *Larus philadelphia - L. ridibundus* | 3.245 |  |
|  |  | *Larus atricilla - L. pipixcan* | 0.675 |  |
|  |  | *Onychoprion anaethetus - O. fuscatus* | 5.913 |  |
|  |  | *Sterna forsteri - S. paradisaea* | 7.505 |  |
|  |  | ***Thalasseus elegans - T. sandvicencis*** | **1.925** | 3 |
|  | Stercorariidae | *Stercorarius pomarinus - S. skua* | 0.32 |  |
|  |  | ***Stercorarius longicaudus - S. parasiticus*** | **6.605** | 3 |
|  | Alcidae | ***Uria aalge - U. lomvia*** | **6.494** | 6 |
|  |  | *Cepphus columba - C. grylle* | 5.452 |  |
|  |  | ***Brachyramphus brevirostris - B. marmoratus*** | **6.527** | 3 |
|  |  | *Synthliboramphus antiquus - S. hypoleucus* | 6.669 |  |
|  |  | ***Aethia cristatella - A. psittacula*** | **5.594** | 3 |
|  |  | ***Fratercula arctica - F. corniculata*** | **1.549** | 3 |
| Columbiformes | Columbidae | *Patagioenas fasciata - P. flavirostris* | 8.556 |  |
|  |  | *Zenaida asiatica - Z. macroura* | 10.909 |  |
|  |  | *Columbina passerina - C. inca* | 6.717 |  |
| Cuculiformes | Cuculidae | *Coccyzus americanus - C. minor* | 5.87 |  |
|  |  | ***Crotophaga ani - C. sulcirostris*** | **2.093** | 7 |
| Strigiformes | Strigidae | *Bubo scandiacus - B. virginianus* | 8.269 |  |
|  |  | *Glaucidium gnoma - G. brasilianum* | 9.998 |  |
|  |  | *Strix occidentalis - S. varia* | 10.559 |  |
|  |  | *Asio flammeus - A. otus* | 9.568 |  |
|  |  | *Aegolius funereus - A. acadicus* | 13.303 |  |
| Caprimulgiformes | Caprimulgidae | *Chordeiles minor - C. acutipennis* | 9.015 |  |
| Apodiformes | Apodidae | *Chaetura pelagica - C. vauxi* | 2.464 |  |
|  | Trochilidae | ***Archilochus alexandri - A. colubris*** | **1.593** | 3 |
|  |  | *Calypte anna - C. costae* | 4.038 |  |
|  |  | *Selasphorus platycercus - S. sasin* | 2.277 |  |
| Coraciiformes | Alcedinidae | *Megaceryle torquata - M. alcyon* | 5.595 |  |
| Piciformes | Picidae | *Melanerpes erythrocephalus - M. formicivorus* | 10.362 |  |
|  |  | *Melanerpes aurifrons - M. carolinus* | 4.625 |  |
|  |  | ***Sphyrapicus nuchalis - S. ruber*** | **0.571** | 1,2 |
|  |  | ***Picoides nuttallii - P. scalaris*** | **0.964** | 2,3,8 |
|  |  | *Picoides arizonae - P. villosus* | 4.178 |  |
| Passeriformes | Tyrannidae | *Contopus cooperi - C. virens* | 10.311 |  |
|  |  | ***Empidonax alnorum - E. traillii*** | **3.486** | 1,2 |
|  |  | *Empidonax hammondii - E. oberholseri* | 12.386 |  |
|  |  | ***Empidonax difficilis - E. occidentalis*** | **1.046** | 1,2 |
|  |  | ***Sayornis nigricans - S. phoebe*** | **2.518** | 2 |
|  |  | *Myiarchus cinerascens - M. crinitus* | 3.835 |  |
|  |  | *Tyrannus couchii - T. dominiscens* | 2.84 |  |
|  |  | *Tyrannus forficatus - T. verticalis* | 2.481 |  |
|  | Laniidae | *Lanius excubitor - L. ludovicianus* | 2.582 |  |
|  | Vireonidae | *Vireo bellii - V. griseus* | 6.218 |  |
|  |  | *Vireo cassini - V. solitarius* | 4.149 |  |
|  |  | *Vireo flavoviridis - V. olivaceus* | 1.446 |  |
|  | Corvidae | ***Cyanocitta cristata - C. stelleri*** | **10.431** | 2,3 |
|  |  | ***Pica hudsonia - P. nutalli*** | **0.576** | 9 |
|  |  | ***Corvus caurinus - C. brachyrhynchos*** | **0.702** | 2 |
|  | Hirundinidae | *Tachycineta bicolor - T. thalassina* | 11.16 |  |
|  |  | *Petrochelidon fulva - P. pyrrhonota* | 3.853 |  |
|  | Paridae | *Poecile carolinensis - P. sclateri* | 5.884 |  |
|  |  | ***Poecile hudsonica - P. rufescens*** | **4.214** | 2 |
|  |  | ***Baeolophus inornatus - B. ridgwayi*** | **3.557** | 1 |
|  |  | ***Baeolophus atricristatus - B. bicolor*** | **0.905** | 1 |
|  | Sittidae | ***Sitta pusilla - S. pygmaea*** | **11.108** | 2 |
|  | Polioptilidae | ***Polioptila californica - P. melanura*** | **2.077** | 2 |
|  | Regulidae | *Regulus calendula - R. satrapa* | 15.191 |  |
|  | Turdidae | *Sialia currucoides - S. mexicana* | 3.862 |  |
|  |  | *Catharus fuscescens - C. minimus* | 1.739 |  |
|  | Mimidae | *Toxostoma curvirostre - T. redivivum* | 6.483 |  |
|  | Motacillidae | *Motacilla alba - M. tschutschensis* | 3.168 |  |
|  |  | *Anthus rubescens - A. spragueii* | 8.622 |  |
|  | Bombycillidae | *Bombycilla cedrorum - B. garrulus* | 8.175 |  |
|  | Parulidae | ***Vermivora chrysoptera - V. pinus*** | **3.392** | 2 |
|  |  | *Vermivora luciae - V. virginiae* | 1.015 |  |
|  |  | *Dendroica pensylvanica - D. petechia* | 3.58 |  |
|  |  | ***Dendroica occidentalis - D. townsendi*** | **0.468** | 1,2 |
|  |  | *Dendroica dominica - D. palmarum* | 3.843 |  |
|  |  | ***Dendroica graciae - D. nigrescens*** | **0.613** | 1,10 |
|  |  | *Dendroica castanea - D. tigrina* | 4.923 |  |
|  |  | *Dendroica cerulea - D. kirtlandii* | 4.295 |  |
|  |  | ***Seiurus motacilla - S. noveboracensis*** | **4.898** | 2 |
|  |  | ***Oporornis philadelphia - O. tolmiei*** | **1.861** | 2 |
|  |  | *Wilsonia canadiensis - W. pusilla* | 5.292 |  |
|  | Emberizidae | ***Pipilo erythrophthalmus - P. maculatus*** | **0.456** | 2 |
|  |  | ***Pipilo alberti - P. crissalis*** | **1.695** | 1 |
|  |  | *Spizella breweri - S. pusilla* | 4.88 |  |
|  |  | *Ammodramus maritimus - A. nelsoni* | 2.696 |  |
|  |  | ***Melospiza georgiana - M. lincolnii*** | **2.939** | 2,3 |
|  |  | *Zonotrichia albicollis - Z. querula* | 2.167 |  |
|  |  | ***Zonotrichia leucophrys - Z. atricapilla*** | **0.304** | 1,11 |
|  |  | *Junco hyemallis - J. phaeonotus* | 0.166 |  |
|  |  | ***Calcarius ornatus - C. pictus*** | **3.655** | 1,3 |
|  | Cardinalidae | *Piranga flava - P. rubra* | 7.776 |  |
|  |  | *Piranga ludoviciana - P. olivacea* | 6.997 |  |
|  |  | *Cardinalis cardinalis - C. sinuatus* | 7.196 |  |
|  |  | *Pheucticus ludovicianus - P. melanocephalus* | 4.887 |  |
|  |  | ***Passerina amoena - P. caerulea*** | **5.108** | 1,2 |
|  |  | ***Passerina ciris - P. versicolor*** | **3.967** | 1,2,3 |
|  | Icteridae | ***Euphagus carolinus - E. cyanocephalus*** | **4.314** | 2,3 |
|  |  | ***Quiscalus major - Q. mexicanus*** | **0.599** | 1,2 |
|  |  | *Molotrhus aeneus - M. ater* | 2.272 |  |
|  |  | *Icterus cucullatus - I. spurius* | 5.248 |  |
|  |  | *Icterus galbula - I. gularis* | 4.522 |  |
|  | Fringillidae | *Carpodacus purpureus - C. cassinii* | 6.137 |  |
|  |  | *Loxia curvirostra - L. leucoptera* | 2.467 |  |
|  |  | *Carduelis lawrencei - C. tristis* | 5.628 |  |

1. Johnson NK, Cicero C (2004) New mitochondrial DNA data affirm the importance of Pleistocene speciation in North American birds. Evolution 58: 1122-1130.
2. Weir JT, Schluter D (2007) The latitudinal gradient in recent speciation and extinction rates of birds and mammals. Science 315: 1574-1576.
3. Tavares ES, Baker AJ (2008) Single mitochondrial gene barcodes reliably identify sister species in diverse clades of birds. BMC Evol Biol 8: 81.
4. Kennedy M, Page RDM (2002) Seabird supertrees: combining partial estimates of Precellariiform phylogeny. Auk 119: 88-108.
5. Austin JJ, Bretagnolle V, Pasquet E (2004) A global molecular phylogeny of the small *Puffinus* Shearwaters and implications for systematics of the little-Audubon's Shearwater complex. Auk 121: 847-864.
6. Thomas GH, Wills MA, Székely (2004) A supertree approach to shorebird phylogeny. BMC Evol Biol 4: 28.
7. Hughes JM (2003) Phylogeny of cooperatively breeding cuckoos (Cuculidae, Crotophaginae) based on mitochondrial gene sequences. Naturwissenschaften 90: 231-233.
8. Weibel AC, Moore WS (2002) Test of a mitochondrial gene-based phylogeny of Woodpeckers (Genus *Picoides*) using an independent nuclear gene, β-Fibrinogen intron 7. Mol Phyl Evol 22: 247-257.
9. Lee S, Parr CS, Hwang Y, Mindell DP, Choe JC (2003) Phylogeny of magpies (genus *Pica*) inferred from mtDNA data. Mol. Phyl. Evol. 29: 250-257.
10. Lovette IJ, Pérez-Emán JL, Sullivan JP, Banks RC, Fiorentino I, *et al*. A comprehensive multilocus phylogeny for the wood-warblers and a revised classification of the Parulidae (Aves). Mol. Phyl. Evol. 57: 753-770.
11. Zink RM, Dittmann DL, Rootes WL (1991) Mitochondrial DNA variation and the phylogeny of *Zonotrichia*. Auk 108: 578-584.
