# Supplementary material for: DNA Barcode Libraries Provide Insight into Continental Patterns of Avian Diversification
Source: PLoS One. 2011 Jul 27;6(7):e20744. doi: 10.1371/journal.pone.0020744 (PMC3144888; doi:10.1371/journal.pone.0020744)
Supplement: Table S3 — List of nearest congeneric neighbours in the dataset from the Palearctic. Taxonomic information for each pair and its genetic distance (K2P) are provided. Pairs identified as sister species are in bold and the references used to identify them are listed. (DOC) [file pone.0020744.s003.doc]

**Table S3**. List of nearest congeneric neighbours in the dataset from the Nearctic. Taxonomic information for each pair and its COI genetic distance (K2P) are provided. Pairs identified as sister species are in bold and the references used to identify them are listed.

| **Order** | **Family** | **Species pair** | **COI genetic distance** | **Reference** |
| --- | --- | --- | --- | --- |
| Anseriformes | Anatidae | Anser anser - A. albifrons | 0.956 |  |
|  |  | Branta canadensis - B. leucopsis | 1.901 |  |
|  |  | **Cygnus cygnus - C. columbianus** | **0.781** | 1 |
|  |  | Tadorna ferruginea - T. tadorna | 4.422 |  |
|  |  | Anas falcata - A. strepera | 1.486 |  |
|  |  | Anas clypeata - A. querquedula | 4.696 |  |
|  |  | Aythya fuligula - A. marila | 1.09 |  |
|  |  | Mergus merganser - M. squamatus | 4.764 |  |
| Galliformes | Phasianidae | Coturnix coturnix - C. japonica | 1.536 |  |
|  |  | **Tetrao parvirostris - T. urogallus** | **3.744** | 2 |
|  |  | **Lagopus lagopus - L. muta** | **4.708** | 2 |
| Gaviiformes | Gaviidae | Gavia immer - G. adamsii | 0.668 |  |
| Podicipediformes | Podicipedidae | Podiceps grisegena - P. cristatus | 6.426 |  |
| Pelecaniformes | Phalacrocoracidae | Phalacrocorax carbo - P. pelagicus | 7.069 |  |
| Ciconiiformes | Ardeidae | Ardeola bacchus - A. ralloides | 5.466 |  |
| Falconiformes | Accipitridae | Circus cyaneus - C. macrourus | 7.369 |  |
|  |  | Circus pygargus - C. aeruginosus | 5.926 |  |
|  |  | Buteo lagopus - B. buteo | 1.142 |  |
|  |  | Aquila chrysaetos - A. clanga | 8.515 |  |
|  | Falconidae | Falco vespertinus - F. amurensis | 1.675 |  |
|  |  | Falco peregrinus - F. rusticolus | 4.212 |  |
| Charadriiformes | Charadriidae | Pluvialis fulva - P. apricaria | 2.895 |  |
|  |  | Charadrius leschenaultii - C. mongolus | 3.122 |  |
|  |  | Charadrius dubius - C. morinellus | 13.9 |  |
|  | Scolopacidae | Tringa nebularia - T. erythropus | 8.088 |  |
|  |  | Tringa glareola - T. stagnatilis | 7.596 |  |
|  |  | Numenius arquata - N. madagascariensis | 4.428 |  |
|  |  | **Calidris canutus - C. tenuirostris** | **7.595** | 3 |
|  |  | Calidris alba - C. minuta | 6.423 |  |
|  |  | Calidris maritima - C. alpina | 7.549 |  |
|  |  | Gallinago megala - G. stenura | 0.609 |  |
|  |  | **Phalaropus fulicarius - P. lobatus** | **5.889** | 3 |
|  | Glareolidae | Glareola nordmanni - G. pratincola | 1.615 |  |
|  | Laridae | Chlidonias leucopterus - C. niger | 1.843 |  |
|  |  | Sterna hirundo - S. paradisaea | 6.046 |  |
|  | Stercorariidae | **Stercorarius longicaudus -S. parasiticus** | **6.241** | 3 |
|  | Alcidae | Cepphus grylle - C. carbo | 5.473 |  |
|  |  | Fratercula arctica - F. cirrhata | 5.389 |  |
| Columbiformes | Columbidae | Columba livia - C. rupestris | 2.153 |  |
|  |  | Columba oenas - C. palumbus | 7.596 |  |
| Strigiformes | Strigidae | Otus lettia - O. sunia | 15.294 |  |
|  |  | Bubo bubo - B. scandiacus | 8.684 |  |
|  |  | Strix aluco - S. uralensis | 7.345 |  |
|  |  | Asio flammeus - A. otus | 9.64 |  |
| Piciformes | Picidae | Dendrocopos canicapillus - D. kizuki | 8.387 |  |
|  |  | Picus canus - P. viridis | 5.513 |  |
| Passeriformes | Laniidae | Lanius bucephalus - L. cristatus | 2.906 |  |
|  | Oriolidae | Oriolus chinensis - O. oriolus | 3.344 |  |
|  | Corvidae | **Pyrrhocorax graculus - P. pyrrhocorax** | **9.389** | 4 |
|  |  | Corvus dauuricus - C. monedula | 3.46 |  |
|  | Alaudidae | Calandrella brachydactyla - C. cheleensis | 7.758 |  |
|  |  | Calandrella acutirostris - C. rufescens | 5.917 |  |
|  | Paridae | Poecile montana - P. palustris | 7.151 |  |
|  |  | Cyanistes caeruleus - C. cyanus | 2.978 |  |
|  | Certhiidae | Certhia brachydactyla - C. familiaris | 2.293 |  |
|  | Cinclidae | **Cinclus cinclus - C. pallasii** | **8.771** | 5 |
|  | Phylloscopidae | Phylloscopus collybita - P. trochilus | 15.228 |  |
|  |  | Phylloscopus humei - P. inornatus | 8.332 |  |
|  | Acrocephalidae | Acrocephalus bistrigiceps - A. melanopogon | 7.759 |  |
|  |  | Acrocephalus agricola - A. dumetorum | 9.503 |  |
|  |  | Acrocephalus palustris - A. scirpaceus | 9.066 |  |
|  |  | Acrocephalus arundinaceus - A. orientalis | 7.555 |  |
|  | Megaluridae | **Locustella certhiola - L. ochotensis** | **6.278** | 6 |
|  |  | **Locustella amnicola - L. fasciolata** | **3.787** | 6 |
|  | Sylviidae | Sylvia atricapilla - S. borin | 12.388 |  |
|  |  | Sylvia communis - S. mystacea | 10.432 |  |
|  | Muscicapidae | Muscicapa dauurica - M. griseistica | 8.819 |  |
|  |  | Ficedula albicilla - F. parva | 5.742 |  |
|  |  | Ficedulla albicollis - F. hyopleuca | 2.015 |  |
|  |  | Ficedula narcissina - F. zanthopygia | 12.263 |  |
|  |  | Oenanthe deserti - O. pleschanka | 8.945 |  |
|  |  | Oenanthe isabellina - O. oenanthe | 6.02 |  |
|  |  | Saxicola insignis - S. rubetra | 8.067 |  |
|  | Turdidae | Monticola gularis - M. saxatillis | 7.961 |  |
|  |  | Turdus obscurus - T. pallidus | 0.933 |  |
|  |  | Turdus naumanni - T. ruficollis | 1.097 |  |
|  |  | Turdus philomelos - T. viscivorus | 9.909 |  |
|  | Prunellidae | Prunella montanella - P. prunella rubida | 5.477 |  |
|  | Motacillidae | Anthus godlewskii - A. richardi | 7.997 |  |
|  |  | Anthus hodgsoni - A. trivialis | 4.158 |  |
|  |  | Anthus petrosus - A. spinoletta | 1.311 |  |
|  | Emberizidae | Emberiza cia - E. godlewskii | 3.08 |  |
|  |  | Emberiza buchanani - E. hortulana | 6.097 |  |
|  |  | Emberiza tristrami -E. variabilis | 3.074 |  |
|  |  | Emberiza aureola - E. rustica | 10.848 |  |
|  |  | Emberiza bruniceps - E. melanocephala | 6.076 |  |
|  | Fringillidae | Fringilla coelebs - F. montifringilla | 8.573 |  |
|  |  | Leucosticte arctoa- L. brandti | 4.295 |  |
|  |  | Carduelis chloris - C. sinica | 4.206 |  |
|  |  | Loxia curvirostra - L. pytyopsittacus | 0.556 |  |
|  |  | **Carduelis cannabina - C. flavirostris** | **4.065** | 7 |
|  |  | Coccothraustes coccothraustes - C. personatus | 10.294 |  |
|  | Passeridae | Passer domesticus - P. hispaniolensis | 2.736 |  |

1. Pointer MA, Mundy NI (2008) Testing whether macroevolution follows microevolution: Are color differences among swans (*Cygnus*) attributable to variation at the MCIR locus? BMC Evol Biol 8: 249.
2. Dimcheff DE, Drovetski SV, Mindell DP (2002) Phylogeny of tetraoninae and other galliform birds using mitochondrial 12S and ND2 genes. Mol Phyl and Evol 24: 203-215.
3. Thomas GH, Wills MA, Székely (2004) A supertree approach to shorebird phylogeny. BMC Evol Biol 4: 28.
4. Ericson PGP, Jansén A, Johansson US, Ekman J (2005) Inter-generic relationships of the crows, jays, magpies and allied groups (Aves: Corvidae) based on nucleotide sequence data. J Avian Biol 36: 222-234.
5. Voelker G (2002) Molecular phylogenetics and the historical biogeography of dippers (*Cinclus*). Ibis 144: 577-584.
6. Drovetski SV, Zink RM, Fadeev IV, Nesterov EV, Koblik EA *et al.* (2004) Mitochondrial phylogeny of *Locustella* and related genera. J Avian Biology 35: 105-110.
7. Arnaiz-Villena A, Moscoso J, Ruiz del Valle V, Gonzalez J, Reguera R, *et al.* (2007) Bayesian phylogeny of Fringillinae birds: status of the singular African oriole finch *Linurgus alivaceus* and evolution and heterogeneity of the genus *Carpodacus*. Acta Zoologica Sinica 53: 826-834.
